# Targeting Key Enzymatic Snake Venom Proteins Using Repurposed Small Molecule Inhibitors: Emerging Adjuncts to Antivenom Therapy

**DOI:** 10.3390/ijms27136000

**Published:** 2026-07-03

**Authors:** Nisha Reghu, S. Kiruthika, Anand Krishna Santhosh, Aparna Lakshmi Narayanan, Krishna Geetha Geetha, Noureen Sidheek, Sai Sahithi Danthuluri, Bipin Gopalakrishnan Nair, Muralidharan Vanuopadath

**Affiliations:** School of Biotechnology, Amrita Vishwa Vidyapeetham, Amritapuri, Clappana PO., Kollam 690 525, Kerala, India

**Keywords:** snakebite envenoming, snake venom, antivenom, small molecule inhibitors, snake venom protease, phospholipase A_2_, next-generation antivenom

## Abstract

Snakebite envenoming is a neglected tropical disease causing approximately 81,410–137,880 deaths globally each year and four-fold more disabilities than mortality rate. Despite the availability of antivenoms for treatment, several in vitro and in vivo preclinical studies have shown that their efficacy is limited by many factors including regional venom variation and poor neutralization of major venom toxins. To address these limitations, the potential of alternatives including aptamers, recombinant monoclonal antibodies, camelid antibodies, small molecule inhibitors, and natural product-based inhibitors targeting key venom proteins have been explored. Among these, small molecule inhibitors targeting key enzymatic snake venom proteins are emerging as adjuvants to antivenom treatment. Most of these small molecules are repurposed drugs with established safety, oral bioavailability, and lower cost compared to antivenoms. In this regard, this review tries to compile available information regarding the use of small molecule inhibitors to counteract envenomation with special emphasis on three major enzymatic snake venom protein families: phospholipase A_2_, snake venom metalloproteinases, and snake venom serine proteases. In vitro studies have shown that these small molecule inhibitors used either alone or in combination with antivenom can potentially reduce the adverse effects of venom-induced coagulopathy, neurotoxicity, tissue damage, and inflammation. However, critical gaps remain including limited human clinical trial data, uncertain efficacy across diverse venoms, and undefined dosing strategies. Overall, small molecules represent a mechanistically targeted and clinically promising adjunct to antivenom therapy, warranting further validation through randomized trials, pharmacokinetic studies, and development of field-applicable treatment protocols.

## 1. Introduction

Snakebite envenoming (SBE) is a neglected tropical disease, and as per World Health Organization reports, 5.4 million people are subjected to snakebites, resulting in approximately 81,410–137,880 deaths globally every year [1]. Among the many factors, lack of effective antivenom, improper identification of bitten snake species, and poor healthcare facilities in rural areas exacerbate the complications. Irrespective of this, timely administration of available antivenom remains the mainstay treatment for snakebite envenoming [2]. They are produced by immunizing large animals, including horses and sheep, using small doses of snake venom and further purifying the antibodies from the plasma of the donor animals. Antivenom can reverse systemic envenoming and is indispensable once the patient reaches hospital care [3,4]. Conventional antivenoms remain the main effective and life-saving intervention in cases of envenomation, neutralizing systemic symptoms and helping to reduce the progression of local damage. Despite their proven efficacy, their use can face challenges such as the risk of adverse reactions, geographical variation in venoms, and differences between production batches [5,6,7,8,9,10,11]. Moreover, antivenom often shows limited ability to reverse local tissue damage and certain presynaptic neurotoxic effects [2,3]. These limitations are especially significant in rural areas, where snakebite cases are more common and where delay in reaching proper medical care often determines the treatment outcome [3].

To overcome these limitations and improve treatment outcomes, there is an urgent need to develop better antivenoms and alternative therapeutic approaches. These efforts should focus on achieving greater clinical effectiveness, wider availability, and improved safety. To streamline effective treatment strategies, a better understanding of snake venom proteins is needed, and this is mostly achieved through conventional bottom-up proteomics [12]. Recombinant monoclonal antibodies and antibody fragments emerge as novel therapeutic options due to their superior specificity, reduced immunogenicity, and capacity of neutralizing medically important toxins [13]. In contrast, recombinant strategies enable selection of broadly neutralizing antibodies targeting toxins from multiple snake species to be designed. Single-domain antibodies (sdAbs), particularly camelid-derived nanobodies, have been widely explored because of their high thermal stability, small size, high tissue penetrability, and increased capacity to bind concealed epitopes which are not accessible using conventional antibodies [14]. Apart from antibody-based therapeutics, there are other strategies which include protein-binding scaffolds such as ankyrin repeat proteins and aptamers which are short single-stranded oligonucleotides with high affinity and specificity and can bind toxin targets [15,16]. In more recent work, these strategies have been found to successfully neutralize key snake venom toxin families, which include phospholipase A_2_, snake venom serine proteases, and snake venom metalloproteases. Additionally, small molecule inhibitors are promising candidates that can effectively inhibit the conserved sites of snake venom toxins, and thus the whole toxin family [17,18]. Small molecule inhibitors have different characteristics in comparison to biological therapeutics; they are chemically stable, resistant to gastrointestinal degradation, and can be administered orally, which can be a benefit for early prehospital intervention in snakebite management. Their rapid tissue distribution, relatively low production cost, and large-scale chemical synthesis offer additional therapeutic potential [19]. This review highlights the recent advances in small molecule inhibitors targeting the major toxins of snake venom and their potential application in next-generation snakebite therapeutics.

## 2. Small Molecule Inhibitors for Snakebite Envenomation

**Figure 1:**
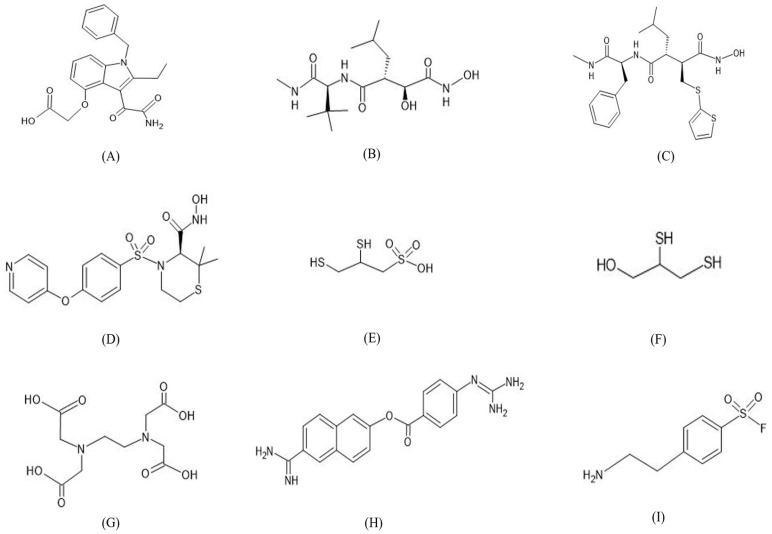
Chemical structures of small molecule inhibitors targeting snake venom toxins. (**A**) Varespladib. (**B**) Marimastat. (**C**) Batimastat. (**D**) Prinomastat. (**E**) 2,3-Dimercapto-1-propanesulfonic acid (DMPS). (**F**) Dimercaprol. (**G**) Ethylenediaminetetraacetic acid (EDTA). (**H**) Nafamostat. (**I**) 4-(2-Aminoethyl)benzenesulfonyl fluoride hydrochloride (AEBSF)/Pefabloc.

**Figure 2:**
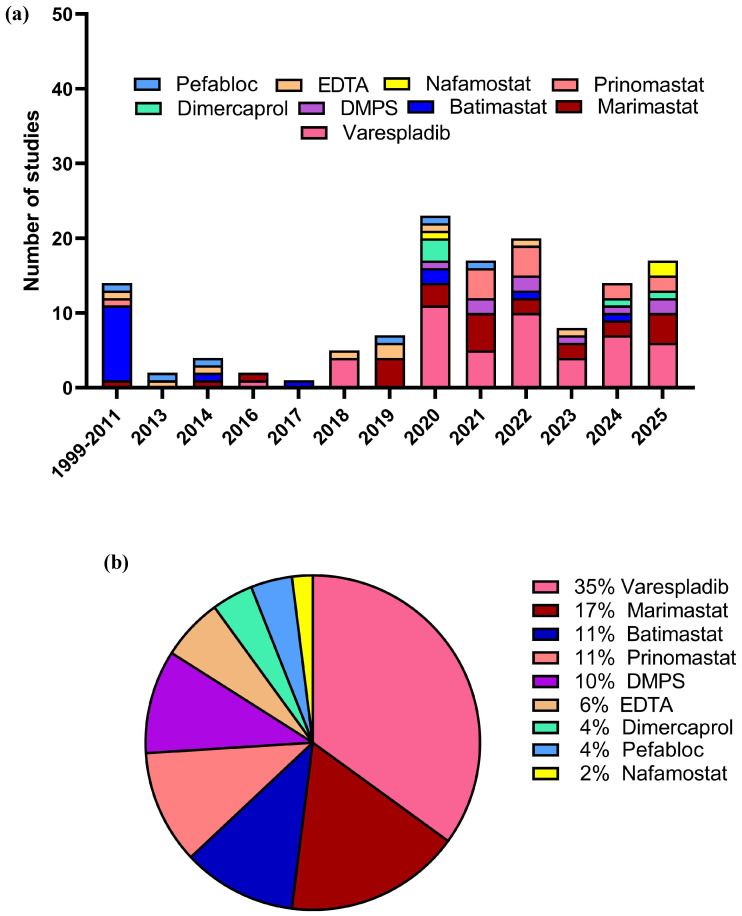
(**a**) Year-wise distribution of studies investigating small molecule inhibitors as adjuncts for antivenom from 1999 to 2025. (**b**) Percentage distribution of small molecule inhibitors used in snakebite therapeutics based on the literature. Varespladib accounted for the highest proportion of studies (35%), followed by marimastat (17%), batimastat (11%), prinomastat (11%), DMPS (10%), and EDTA (6%). Other inhibitors, pefabloc and dimercaprol, each accounted for 4% of the reports, while nafamostat represented 2% of the total number of studies.

Small molecule inhibitors (SMIs) are low-molecular-weight compounds that directly block key venom toxin activities, usually by binding catalytic metal ions or active sites within conserved toxin families. In snakebite envenomation, they are being explored as adjuncts or alternatives to antivenom because as mentioned earlier, they may be less costly to manufacture, easier to formulate, and in some cases suitable for early community or pre-referral use before a patient reaches hospital care. They can effectively inhibit major enzymatic snake venom proteins such as phospholipase A_2_ (PLA_2_), snake venom metalloproteases (SVMPs), and snake venom serine proteases (SVSPs), which are associated with major clinical manifestations such as coagulopathy, hemorrhage, tissue necrosis, and systemic toxicity [20]. This review highlights selected SMIs (Figure 1) and focuses on studies describing how these agents act against PLA_2,_ SVMP, and SVSP protein families from medically important snake species. Multiple repurposed SMIs are examined for their new therapeutic applications, such as varespladib as a PLA_2_ inhibitor [21]; unithiol (DMPS), marimastat, batimastat, prinomastat, tanomastat, EDTA, and dimercaprol as SVMP inhibitors [22]; and pefabloc and nafamostat as SVSP inhibitors [23]. Figure 2a illustrates the year-wise distribution of studies investigating small molecule inhibitors for snakebite treatment from 1999 to 2025. Among these SMIs, our analysis has indicated that varespladib represented the highest proportion of studies (35%), followed by marimastat (17%), batimastat (11%), prinomastat (11%), DMPS (10%), and EDTA (6%). Other inhibitors, such as pefabloc and dimercaprol, each accounted for 4% of the reports, while nafamostat represented 2% (Figure 2b and Table 1). Studies have also shown that the combination of SVMP and PLA_2_ inhibitors, marimastat and varespladib, can effectively neutralize multiple toxin families present in viper venom, highlighting their potential as complementary therapeutic agents for snakebite treatment [24].

### 2.1. Varespladib

Varespladib has emerged as one of the most promising small molecule inhibitors targeting PLA_2_ snake venom proteins and has been tested more broadly and successfully across different snake species than many other SMIs (Figure 1A, Table 1). Originally developed for treating inflammatory cardiovascular indications in humans [25], it was later repurposed to target snake venom PLA_2_s which mediates inflammatory, myotoxicity, anticoagulation, and neurotoxicity symptoms post-envenomation [26]. Its orally bioavailable prodrug, methyl-varespladib, makes it especially attractive as a pre-referral treatment, and this has led to the fastest progression of varespladib toward clinical application [26,27]. Although the BRAVO Phase II clinical trial did not demonstrate significant improvement in the primary outcome in the entire study population, patients who received treatment earlier showed indications of better clinical response. These findings have strengthened interest in rapid administration of varespladib following envenomation [28]. Varespladib is therefore presented as the most clinically advanced PLA_2_-targeted SMI and a central component of combination strategies with SVMP inhibitors such as marimastat [26].

Varespladib binds in the conserved active site region of snake venom PLA_2_s and blocks access of the phospholipid substrate to the catalytic machinery. In enzymatically active Asp49 PLA_2_s, the drug is thought to occupy the hydrophobic substrate channel and extend into the calcium-binding catalytic site, where it interacts with conserved residues such as His48, Asp49, Tyr52, and Asp99 [25]. Since PLA_2_ catalysis depends on this conserved pocket and on calcium-assisted hydrolysis of the sn-2 bond of membrane phospholipids, active site occupation prevents substrate binding and enzyme function. Functionally, this means varespladib prevents phospholipid hydrolysis, so the venom cannot efficiently generate the lipid products that drive membrane damage, myotoxicity, and other downstream toxic events mediated by PLA_2_-rich venoms [25,26].

The ability of varespladib to neutralize the myotoxic and other effects of PLA_2_-like proteins is an intriguing property, as these proteins are structurally classified as Group II PLA_2_s but contain a Lys49 residue in place of Aslp49. This modification prevents the coordination of the calcium ion cofactor, making these proteins incapable of performing phospholipolysis. Thus, their biological activities are entirely independent of catalysis [29]. Recent studies demonstrated that varespladib effectively inhibits these non-catalytic homologs by mechanically restricting their access to cell membranes. Structural analysis reveals that the inhibitor binds within the hydrophobic channel, which is stabilized by hydrogen bonds with key residues like His48 and Lys49, and it can also occupy functional regions such as the membrane-disrupting site and membrane-docking site. This binding halts the destabilization of membranes that leads to cell death. Thus, varespladib acts as a multifunctional inhibitor capable of neutralizing both the catalytic activities of Asp49-PLA_2_s and the non-catalytic effects of Lys49-PLA_2_-like proteins [30].

### 2.2. Marimastat

Marimastat is a synthetic peptidomimetic hydroxamate that was originally developed as a matrix metalloprotease inhibitor for cancer and has become one of the best-supported SMIs in snakebite research [31]. Its relevance to inhibition lies in its potent inhibition of zinc-dependent snake venom metalloproteases, which are responsible for hemorrhage, venom-induced consumption coagulopathy, and local tissue damage [32]. Its potential for clinical translation is further supported by its good solubility and oral bioavailability, making it more suitable for practical use compared to other compounds from the same class. Its key inhibitory step is zinc chelation in the active site. The hydroxamate group of marimastat coordinates the catalytic Zn^2+^ directly in the metalloprotease active site, thereby disrupting the zinc-dependent catalytic machinery required for peptide bond hydrolysis. This prevents activation of the catalytic water molecule and blocks substrate cleavage, ultimately inhibiting the proteolytic activity of snake venom metalloproteases [33].

### 2.3. Batimastat

Batimastat is a first-generation hydroxamate metalloprotease inhibitor and one of the earliest synthetic compounds to provide proof-of-concept that direct inhibition of SVMPs could reduce snakebite-related complications [34]. It was first developed as a human MMP inhibitor to treat cancer, but in animal models of SBE it showed that blocking SVMPs could reduce local hemorrhage, dermo necrosis, and systemic toxicity caused by viper venoms [20]. The mechanism involves batimastat’s hydroxamate group coordinating the catalytic zinc ion in the SVMP active site, acting as a transition-state mimic of peptide substrate hydrolysis, similar to marimastat, as both are hydroxamate-based peptidomemetic inhibitors [35]. This made batimastat important for establishing SVMPs as druggable venom targets. However, despite experimental proofs, reports indicate that batimastat has weaker translational prospects than marimastat because of bioavailability and the need for parenteral administration [36].

### 2.4. Prinomastat

Prinomastat (AG-3340) is a hydroxamate-based matrix metalloprotease inhibitor that targets SVMPs by binding to the catalytic site and chelating the active site Zn^2+^ ion, thereby blocking the metalloprotease-dependent hydrolysis required for venom proteolytic activity [37]. Owing to the structural and functional similarity between mammalian matrix metalloproteases and snake venom metalloproteases, prinomastat has been considered a potential repurposable therapeutic for snakebite envenoming [38].

### 2.5. (Dimercaptopropanesulfonic Acid) DMPS

DMPS is also known as unithiol and is a water-soluble thiol-chelating agent that targets snake venom metalloproteases through Zn^2+^ chelation at the enzyme active sites, where its two thiol groups (-SH) bind to zinc with high affinity, forming stable chelate complexes that remove the metal ion from the SVMP catalytic site, rendering the enzyme inactive. This nonspecific chelation mechanism explains why DMPS has lower potency than hydroxamate inhibitors. A recently completed Phase I open-label dose-escalation safety trial in Kenya with 64 healthy adult volunteers demonstrated no dose-limiting toxicities or serious adverse events, showing promising pharmacokinetic properties [39,40].

### 2.6. Dimercaprol

Dimercaprol or British anti-Lewisite is another metal chelator that has been repurposed experimentally to inhibit SVMPs [41]. It acts as a metal chelator by binding to the zinc ions (Zn^2+^) required for SVMP catalytic activity through its two thiol (-SH) groups. The thiol-based chelation removes the zinc ion from the SVMP active site, rendering the enzyme inactive since zinc is essential for metalloprotease function [42].

### 2.7. EDTA

EDTA is a metal chelator that inhibits zinc-dependent snake venom metalloproteases by removing the metal ion required for their catalytic activity. Under experimental conditions, EDTA reduced hemorrhage, dermonecrosis, fibrinogen consumption, and other SVMP-driven effects, particularly in *Bothrops* models, confirming the contribution of SVMPs in local tissue damage and coagulopathy [34].

### 2.8. Nafamostat

Nafamostat or Nafamostat mesilate (FUT-175) is a synthetic serine protease inhibitor that has recently gained attention because of its ability to inhibit SVSPs associated with venom-induced coagulation disturbances. In trypsin-like serine proteases, it acts as a slow tight-binding substrate that forms a covalent acyl enzyme intermediate. This enzyme cleaves nafamostat, releasing 6-amidino-2-napthol, while 4-guanidinobenzoic acid remains covalently attached to the catalytic serine; the very slow deacylation rate prolongs inhibition. Since snake venom serine proteases are trypsin-like enzymes, this mechanism is likely to explain their inhibition by nafamostat as well [43]. Although serine proteases have received less attention than other inhibitors targeting SVMPs or PLA_2_ toxins, recent in vitro studies have demonstrated that nafamostat can effectively inhibit SVSP activity in venoms from species such as *Bitis arietans*, *Bitis gabonica*, and *Causus rhombeatus* [44]. However, its role in snakebite management remains experimental and compared with other inhibitors, nafamostat is still considered as an emerging rather than a clinically established SMI.

### 2.9. Pefabloc

Commonly known as AEBSF, pefabloc is a serine protease inhibitor that has been mainly used in snakebite research to understand venom mechanisms rather than as a direct therapeutic agent.

**Table 1:** Studies using small molecule inhibitors targeting snake venom metalloproteases, snake venom serine proteases, and phospholipase A_2_.

Sl No.	Snake Species	Snake Family	Venom Source/Geographical Location	Small Molecule Used	Snake Venom Protein(s) Targeted	Study Type (In Vitro/In Vivo/Ex Vivo/Clinical)	Inference from the Study	Year of Study	Reference
1	*N. naja*, *N. atra*, *N. nigricolis*, *N. sputatrix*, *B. asper*, *C. oreganus*, *C. basilicus*, *N. annulifera*, *N. melanoleuca*, *N. nivea*, *C. rhombeatus*, *E. carinatus*, *M. xanthina*, *A. c. laticinctus*, *T. stejnegeri*, *B. arietans*, *D. russelli*, *C. atrox*, *B. gabonica*	Elapidae and Viperidae	Venomtech Ltd., Sandwich (UK), Latoxan Valence (France), Sigma Aldrich, Poole (UK), Kentucky Zoo, Liverpool School of Tropical Medicine, Liverpool (UK).	Prinomastat and marimastat	SVMP	In vitro	Prinomastat is more effective than marimastat at inhibiting low levels of metalloprotease activity in cobra. Both inhibitors completely inhibit fibrinogenolytic activity. Both the drugs show better activity with viper venom than elapid venom.	2025	[31]
2	*B.arietans*, *B.gabonica*, *H.haemachatus*, *N.mossambica*, *N. nivea*, *C. rhombeatus*, *D angusticeps*, *D polylepis*	Elapidae and Viperidae	Limpopo Province of South Africa	Varespladib, marimastat, dimecaprol, nafamostat	PLA_2_, SVMP, SVSP	In vitro	Varespladib potently inhibited PLA_2_ activity, marimastat inhibited SVMPs, and nafamostat strongly inhibited SVSPs across multiple species. Dimercaprol is a SVMP inhibitor, but with lower potency than marimastat.	2025	[44]
3	*P. papuanus*	Elapidae	Papua New Guinea, Indonesian Papua, and Australia’s Torres Strait Islands	Varespladib	PLA_2_	In vitro, In vivo	In this study, varespladib inhibited PLA_2_-induced lung injury caused by the venom of *Pseudechis papuanus* in mice.	2025	[45]
4	*N. atra*	Elapidae	Huangshan Snake Hacienda (Huangshan, China)	Varespladib	PLA_2_	In vitro, In vivo	Varespladib reduced *N. atra*-induced acute liver injury by inhibiting PLA2 activity and also suppressing Nrf2-mediated ferroptosis, mitochondrial dysfunction, excessive mitophagy, and mitochondria-dependent apoptosis.	2025	[46]
5	*D. Russelii*	Viperidae	Different locations in India, Andhra Pradesh, Goa, Karnataka (KA), Kerala (KL), Madhya Pradesh (MP), Maharashtra (MH), Punjab (PB), Rajasthan (RJ), Tamil Nadu (TN), West Bengal (WB)	Varespladib, marimastat, nafamostat	PLA_2_, SVMP, SVSP	In vitro, In vivo	Combined marimastat and varespladib reduced PT and aPTT alterations, inhibited key venom enzymes, and reduced venom toxicity. The combination provided protection against geographically diverse *D. russelii* venom in mice, even when treatment was delayed.	2025	[47]
6	*C. culminatus*	Viperidae	Instituto de Biotecnología, UNAM	Prinomastat, marimastat, DMPS	SVMP	In vitro	Significant ontogenetic and geographic variation with venom was observed: commercial Mexican antivenoms could neutralize the procoagulant activity in in vitro conditions alone. Marimastat and prinomastat inhibited venom activity, supporting their potential as adjunct treatment for snakebite envenomation.	2025	[48]
7	*Bothrops* spp., *Bitis* spp., *Crotalus* spp., *Daboia* spp., *C. rhodostoma*, *D. typus*	Viperidae	Clinical trial in Kenya, Asia, Africa, North America, and South America	Unithiol (DMPS)	SVMP	In vitro, In vivo and Clinical	Unithiol was safe and tolerated in humans. Only mild issues like metallic taste or nausea were reported. It showed good absorption. It is affordable and thermally stable. Unithiol offers potential as a field treatment for snakebites.	2025	[40]
8	*M. fulvis*	Elapidae	Florida, USA	Varespladib	PLA_2_	Clinical	Varespladib treatment led to a complete and fast recovery, reduced the need for mechanical ventilation, and decreased hospitalization time with minimal adverse effects.	2025	[49]
9	*A. contortrix*, *C. helleri Type B*, *N. atra*, *N. mossambica*, *N. nigricollis*, *V. ammodytes*	Viperidae and Elapidae	South Carolina, USA, China, South Africa, West Africa, Europe	Marimastat, varespladib	SVMPs, PLA_2_	In vitro	Study demonstrated that marimastat worked against *D. russelii* venom while varespladib mitigated the toxicity of *N. naja* venom, and their combination provided broad protection.	2025	[50]
10	*P. australis*, *P. porphyriacus*, *P. colletti*, *P. guttatus*, *P. papuanus*, *P. butleri*, *E.coronatus*, *S. suta*, *S. punctata*, *S. fasciata*, *A. superbus*, *A. labialis*, *A. ramsayi*, *A. antarcticus*, *A. pyrrhus*, *D. maculata*, *D. devisi*, *P. textilis*, *P. guttata*, *P. modesta*, *N. scutatus*, *O. scutellatus*, *C. nigrescens*, *C. pallidiceps*, *D. psammophis*, *D. vestigata*, *E. curta*, *H. bitorquatus*, *H. stephensii*, *H. bungaroides*, *S. flagellum*, *S. monachus*, *T. carinatus*, *and V. annulata*	Elapidae	Venom Supplies, Tanunda, South Australia	Varespladib	PLA_2_	In vitro	Anticoagulant activity is more widespread among Australasian elapids, and it is driven by high levels of PLA_2_. Both commercial Tiger Snake Antivenom (TSAV) and varespladib neutralized these anticoagulant effects.	2024	[51]
11	*E. ocellatus*, *C. atrox*, *B. arientans*, *C. rhodostoma*, *B. jararaca*	Viperidae	Nigeria, US, Malayasia, Brazil	Marimastat, prinomastat, DMPS, dimercaprol	SVMP	In vitro, In silico	The first high-throughput screening for snake venom identified 14 inhibitors and four novel SVMP inhibitors with nanomolar potency comparable to marimastat. CTS-1027 and prinomastat were considered promising lead compounds for snakebite treatments.	2024	[52]
12	*C. atrox*	Viperidae	University of Northern Colorado (UNC) Animal Facility, USA	Marimastat	SVMP	In vitro	Marimastat directly attached to and stabilized SVMP toxins, confirming its specificity. The study also ensured the use of PISA/TPP as effective high-throughput approaches for inhibitor–venom interactions.	2024	[53]
13	*B. lanceolatus*	Viperidae	Martinique	Batimastat	SVMP	In vitro, In vivo	Batimastat did not prevent thrombosis, showing that metalloproteinases are not the primary drivers of the observed effect.	2024	[54]
14	*A. picadoi*, *B. asper*, *C. godmani*, *C. petlalcalensis*, *C. tzotzilorum*, *C. wilsoni*, *M. mexicanus*, *M. nummifer*, *M. occiduus*, *M. olmec*	Viperidae	Costa Rica, Siltepec Chiapas (Mexico), San Andres Tenejapan, Veracruz (Mexico), San Cristobal Chiapas (Mexico), Honduras, Mapastepec Chiapas (Mexico)	Varespladib	PLA_2_	In vitro, Ex vivo	Varespladib neutralized the effects of some species which were PLA_2_ -driven, and it was ineffective against non-PLA_2_ -mediated toxicity. The ICP antivenom showed partial or poor protection against several species.	2024	[55]
15	*N. nigricollis*, *N. pallida*	Elapidae	Tanzania and Nigeria	Varespladib	PLA_2_	In vitro, In vivo	Local injection of varespladib inhibited venom-mediated dermonecrosis, becoming a therapeutic approach that can be used for snakebite-related tissue damage in rural regions of Africa.	2024	[56]
16	*Thai D. siamensis*, *Javanese D. siamensis*	Viperidae	Thailand, Australia	Varespladib	PLA_2_	In vitro	Fraction 8 was found to be a major neurotoxic PLA_2_ component. Varespladib and monovalent antivenom neutralized the neurotoxic effects of fraction 8. The neurotoxic effects of combined fraction 8 and fraction 10 was reduced by administering varespladib within 60 min of envenomation.	2024	[57]
17	*T. gumprechti*, *T. macrops*, *T. popeiorum*, *T. trigonocephalus*, *T. vogeli*, *T. barati*, *T. stejnegeri*, *T. sumatranus*, *T. albolabris*	Viperidae	Latoxan (France), Indonesia, China, Taiwan, Queen Saovabha Memorial Institute, Bangkok (Thailand)	Varespladib	PLA_2_	In vitro	The antivenom neutralized the hemorrhagic and procoagulant effects, but it did not affect PLA_2_ activity. Varespladib neutralized the PLA_2_ activity of all venoms.	2024	[58]
18	*D. russelii*, *B. arietans*, *N. nigricollis*, *C. atrox*, *N. naja*, *C. durissus terrificus*	Viperidae and Elapidae	Sri Lanka, Nigeria, Tanzania, United States, captive-bred, Brazil	Varespladib	PLA_2_	In vitro	The study found new PLA_2_ inhibitors besides varespladib through high-throughput screening. However, varespladib remained the most potent inhibitor.	2024	[59]
19	*D. russelii*, *N. naja*	Viperidae and Elapidae	USA	Varespladib, prinomastat	PLA_2_, SVMP	In vitro	Prinomastat and varespladib reduced viper venom damage, but cobra-venom-induced atrophy remained largely unaffected. Antivenom helped only partially to alleviate the pain.	2024	[60]
20	*D. russelii*, *A. contortrix*, *C.atrox*, *C. horridus*, *C. willardi*, *S. miliarus*, *N. naja*, *N. kaouthia*, *H. hypnale*, *E. carinatus*	Viperidae and Elapidae	Multiple sites in India and the USA	Varespladib	PLA_2_	Clinical	Oral varespladib was found to be significant in snakebite treatment when administered within 5 h of envenomation, supporting further validation in Phase III trials.	2024	[61]
21	*D. siamensis*	Viperidae	Yunnan province, China	Varespladib	PLA_2_	In vitro	Antivenom is largely ineffective at reversing established pre-synaptic neurotoxicity and myotoxicity. However, varespladib prevented further damage and partially restored function, extending the therapeutic approach for antivenom administration.	2023	[62]
22	*B. arietans*, *B. asper*, *C. atrox*, *C. rhodostoma*, *D. russelii*, *E. carinatus*, *E. ocellatus*, *N. haje*, *East African N. nigricollis*, *West African N. nigricollis*, *N. pallida*	Viperidae	Nigeria, Costa Rica, USA, Malaysia, Sri Lanka, India, Uganda, Tanzania	DMPS, varespladib, marimastat	PLA_2,_ SVMP	In vitro, In vivo	Combinations of DMPS + varespladib and marimastat + varespladib effectively stopped the dermonecrotic effects, even when administered 1 h after the bite.	2023	[63]
23	*O. scutellatus*	Elapidae	Papua New Guinea, Australia	Varespladib methyl and sodium salt of varespladib	PLA_2_	In vivo	Varespladib and methyl-varespladib reversed severe weakness and prevented mortality, remaining effective beyond the therapeutic activity of antivenom.	2023	[64]
24	*B. pirajai*	Viperidae	Atlantic Forest in southeastern Bahia state, Brazil	Varespladib	PLA_2_	Ex vivo	Varespladib neutralized the neuromuscular blocking effects induced by PLA_2_ and PLA_2_-like toxins (PrTX-I), and directly interacted with muscle-damaging toxins.	2023	[29]
25	*B. pictus*	Viperidae	Peru	Marimastat, EDTA	SVMP	In vitro, In vivo	Marimastat and EDTA effectively inhibited the enzymatic activity of Pic-III, neutralizing its hemorrhagic activity and preventing toxin-induced mitochondrial dysfunction and cytokine secretion.	2023	[65]
26	*R. subminiatus*	Viperidae	Liverpool School of Tropical Medicine	Prinomastat, marimastat, DMPS	SVMP, SVSP	In vitro	SVMP-mediated activation of multiple clotting factors was inhibited more effectively by prinomastat and marimastat than DMPS.	2022	[66]
27	*D. typus*	Viperidae	Sub-Saharan Africa	Marimastat, prinomastat, DMPS, dimercaprol	SVMP, SVSP	In vitro, In vivo	Marimastat showed superior efficacy, while DMPS prolonged survival and showed partial in vivo protection, yet both could be utilized as early treatment options for SVMP-driven coagulopathy.	2022	[67]
28	*M. corallinus*	Elapidae	Brazil	Varespladib	PLA_2_	In vivo	Varespladib alone reduced neurotoxicity and lymphocytosis, while its combination with antivenom showed enhanced activity against hepatotoxicity, nephrotoxicity, and myotoxicity.	2022	[68]
29	*N. naja*, *H. haemachatus*, *D. polylepis*, *D. angusticeps*, *N. pallida*, *N.nigricollis*, *N. haje*	Elapidae	Liverpool School of Tropical Medicine, UK	Varespladib, marimastat	PLA_2_, SVMP	In vitro	PLA_2_ exhibits the most potent anticoagulant activity in cobra species. Varespladib effectively neutralized this activity in cobras, while marimastat showed only limited efficacy.	2022	[69]
30	*C. d. terrificus*	Viperidae	São Paulo, Brazil	Varespladib (crotoxin)	PLA_2_	In vitro, ex vivo	Varespladib is highly effective in preventing the neuromuscular blockade and acts synergistically with antivenom. It is largely effective even when administered after venom exposure.	2022	[70]
31	*B. asper*, *C. d. cumanensis*	Viperidae	Colombia	Varespladib and CP471474	PLA_2_, SVMP	In vivo	Varespladib and CP471474 partially inhibited lethality, myotoxicity, edema, and hemorrhage, with improved efficacy when used together.	2022	[71]
32	*L. muta rhombeata*	Viperidae	Brazil	Varespladib	PLA_2_	In vitro, In vivo	Varespladib reduced PLA_2_ activity and prevented venom-induced coagulation disturbances, particularly when combined with antivenom. However, varespladib did not reduce SVMP-driven haemorhage.	2022	[72]
33	*M. dumerilii carinicauda*	Elapidae	Colombia and Venezuela	Varespladib and coral snake antivenom	PLA_2_	In vitro, In vivo	Combination of antivenom and varespladib delivered partial protection against coagulopathy, leukocytosis, and nephrotoxicity, while neither antivenom nor varespladib alone prevented systemic toxic effects.	2022	[73]
34	*B.sindanus*, *B.caeruleus*, *B.multicintus*, *B. candidus*, *B. fasciatus*	Elapidae	Sindh Province (Pakistan), VINS Bioproduct of India (Tamil Nadu), Taiwan Island, Queen Saovabha Memorial Institute (Thailand), Peninsular Malaya of Malaysia	Varespladib	PLA_2_	In vivo	Varespladib rescued acute neurotoxic effects inhibited by *Bungarus* species venom, but the efficacy was dose-dependent.	2022	[74]
35	*D. acutus*, *A. halys*, *B. multicinctus*, *N. atra*	Viperidae and Elapidae	Huangshan Snake Farm (Huangshan, China)	Varespladib	PLA_2_	In vitro, In vivo	Varespladib is an effective broad-spectrum inhibitor of PLA2 that significantly reduces pathological damage caused by both viperid and elapid venoms.	2022	[75]
36	*P. australis*, *P. butleri*, *P. colletti*, *P. guttatus*, *P. papuanus*, *P. pailsei*, *P. rossignoli*, *P. porphyriacus*, *P. weigeli*	Elapidae	Australia	Varespladib	PLA_2_, SVMP	In vitro	Anticoagulant action for *Pseudechis* venoms is shown by the inhibition of Factor Va. Varespladib effectively neutralized the anticoagulant toxicity, highlighting its potential where existing antivenoms are ineffective.	2022	[76]
37	*P. australis*, *P. butleri*, *P. colletti*, *P. guttatus*, *P. papuanus*, *P. rossignoli*, *P.* sp. *(NT)*	Elapidae	Australia and New Guinea	Varespladib, prinomastat	PLA_2_, SVMP	In vitro	Varespladib significantly inhibited the anticoagulant activity across most *Pseudechis* species, while prinomastat inhibited five out of the seven species, excluding *P. coletti* and *P. rossignolii* venoms. This indicates that metalloprotease inhibitors offer cross-neutralization of PLA_2_-mediated toxins.	2022	[77]
38	*B. atropos*, *B. caudalis*, *B. cornuta*	Viperidae	Drakensberg, Rosh Pinah (Namibia), Kleinsee and Springbok (South Africa)	Varespladib, prinomastat, marimastat	PLA_2_, SVMP	In vitro	Varespladib neutralized anticoagulant activity in *Bitis* spp. venom, while metalloprotease inhibitors were less effective. None of the SMIs neutralized the pseudo-procoagulant activity of *B. caudalis*.	2022	[78]
39	*A. ceratophora*, *A. chlorechis*, *A. desaixi*, *A. nitschei*, *A. squamigera*, *C.cerastes*, *C. gasperettii*, *C. vipera*, *P. superciliaris*	Viperidae	Tanzania, Ghana, Kenya, Burundi, Sub-Saharan Africa, Tunisia, Saudi Arabia, Egypt, Mozambique	DMPS, prinomastat, and marimastat	SVMP	In vitro	Marimastat exhibited the most potent neutralization against the procoagulant effects of all tested venoms compared to prinomastat and DMPS.	2022	[79]
40	*E. ocellatus*, *B. arietans*, *B. asper*, *Daboia* and *Crotalus* genera	Viperidae	West African saw-scaled viper, puff adder	Unithiol (DMPS)	SVMP	In vitro, In vivo, Clinical	Unithiol was safe, well-tolerated, and reached plasma levels high enough to inhibit SVMP in vitro against various viper venoms. It supports further Phase II efficacy trials to use it as a repurposed drug for treating snakebites.	2022	[80]
41	Genera: *Agkistrodon*, *Bungarus*, *Crotalus*, *Daboia*, *Echis*, *Hypnale*, *Micrurus*, *Naja*, and *Sistrurus*	Viperidae	Sixteen geographically diverse centers across the United States and India	Methyl-varespladib	PLA_2_	Clinical	Early use of varespladib, within 5 h, was more effective and improved recovery outcomes and safety profile.	2022	[28]
42	*A. picadoi*, *C. godmani*, *C. tzotzilorum*, *C. wilsoni*, *M. mexicanus*, *M. nummifer*, *M. occiduus*, *M. olmec*, *P. dunni*, *P. lansbergii*, *P. nasutum*, *P. hespere*, *P. ophryomegas*, *P. porrasi*, *P. volcanicum*, and *P. yucatanicum*	Viperidae	Mexico (various regions), Costa Rica, Honduras, and Colombia	DMPS, prinomastat, and marimastat	SVMP	In vitro	ICP antivenom effectively neutralized anticoagulant and pseudo-procoagulant effects in different species. Prinomastat and marimastat showed stronger inhibition against *P. volcanicum* procoagulant toxins compared to ICP and DMPS.	2022	[81]
43	*C. culminatus*, *C. mictlantecuhtli*, *C. molossus*, *C. tzabcan*	Viperidae	Mexico	Prinomastat pefabloc, DMPS	SVMP, SVSP	In vitro	Metalloprotease-driven Factor X activation was more effectively inhibited by prinomastat than DMPS. Conversely, the pseudo-procoagulant activity driven by kallikrein-type serine proteases was selectively inhibited by AEBSF.	2021	[82]
44	*P. mucrosquamatus*	Viperidae	Yangmingshan mountain, Taipei, Taiwan	Varespladib	PLA_2_	In vitro, In vivo	Varespladib significantly inhibited PLA_2_ activity and delayed venom-induced lethality in a rodent model. However, the drug showed limited efficacy against local hemorrhage.	2021	[83]
45	*N. nigricollis*	Elapidae	Nigeria	Varespladib, marimastat	PLA_2_ and SVMP	In vitro	Anticoagulant activity of *N. nigricollis* venom is primarily mediated by basic PLA_2_s rather than the more abundant three-finger toxins. Varespladib effectively neutralized the venom’s anticoagulant effects, whereas marimastat showed limited activity.	2021	[84]
46	*B. moojeni*	Viperidae	CETA – Morungaba, São Paulo	Varespladib	PLA_2_	In vitro, In vivo	Varespladib inhibited MjTX-I activity comparable to its inhibition of PLA_2_ and PLA_2_-like toxins.	2021	[30]
47	*P.affinis*, *P.aspidorhyncha*, *P.inframacula*, *P.textilis*, *P.nuchalis*	Elapidae	South and North Australia	Varespladib	PLA_2_	In vitro	Varespladib inhibited PLA_2_-mediated vasorelaxation and sympatholytic effects of brown snake venoms.	2021	[85]
48	*N. ashei*, *N. katiensis*, *N. mossambica*, *N. nigricincta*, *N. nigricollis*, *N. nubiae*, *N. pallida*	Elapidae	Sub-Saharan Africa	Varespladib, prinomastat, marimastat	PLA2, SVMP	In vitro	Varespladib neutralized the PLA_2_-driven effects of African spitting cobras. Prinomastat showed higher cross neutralization of PLA_2_ toxins than marimastat.	2021	[86]
49	27 venom samples from the genera *Daboia* (*D. russelii*, *D. siamensis*), *Macrovipera* (*M. lebetina turanica*, *M. schweizeri*), *Montivipera* (*M. albizona*, *M. bulgardaghica*, *M. raddei*, *M. wagneri*, *M. xanthina*), and *Vipera* (*V. ammodytes*, *V. aspis*, *V. berus*, *V. kaznakovi*, *V. latastei*, *V. nikolskii*, *V. renardi*, and *V. transcaucasiana*)	Viperidae	Palearctic region, including Europe, Central Asia, and the Near and Middle East. Specific countries/localities include Pakistan, Taiwan, Turkmenistan, Greece, Turkey, Croatia, Slovenia, Montenegro, Bulgaria, France, Italy, Norway, Spain, and Russia	Prinomastat, DMPS	SVMP	In vitro	Prinomastat effectively inhibited metalloprotease-driven Factor X activation in procoagulant venoms of vipers, whereas DMPS failed to neutralize any tested venom activity.	2021	[87]
50	*M. lebetina cernovi*, *M. lebetina obtusa*, *M. lebetina turanica*, *M. schweizeri*, *D.russelii (used for comparison)*	Viperidae	Kazastan, Azerbaijan, Turmenistan (Uzbekistan), Greece, Pakistan	Marimastat and prinomostat	SVMPs	In vitro	Macrovipera venoms induce coagulopathy through the activation of Factor X. Prinomastat showed greater inhibition of this activity than marimastat.	2021	[88]
51	*N. scutatus*, *C. durissus terrificus*, *B. multicinctus*, *O. scutellatus*	Elapidae and Viperidae	Venom Supplies Pty Ltd (Australia), Instituto Butantan (Brazil), Latoxan (France), University of Melbourne (Papua New Guinea)	Varespladib (LY315920), methyl-varespladib (LY333013)	PLA_2_	In vivo	Varespladib and its oral prodrug, methyl-varespladib, delayed or completely prevented lethality from various medically negligible snake venoms when established in mice models. It is also capable of reversing paralysis in severely envenomed mice.	2020	[27]
52	*E. carinatus*, *E. ocellatus*, *B. arietans*, *D. russelii*	Viperidae	India, Nigeria, Sri Lanka	Marimastat, varespladib, dimercaprol, DMPS	SVMP, PLA_2,_ CTLs	In vitro	Marimastat showed potent and broad-spectrum neutralization of procoagulant toxins. Varespladib effectively inhibited most anticoagulant activities. DMPS and dimercaprol were less effective. Combination of varespladib and marimastat provided a broad-spectrum protection against viper envenomation.	2020	[22]
53	*B. asper*, *C. rhodostoma*, *D. acutus*, *D. russelii*, *E. carinatus*, *E. ocellatus*, *O. scutellatus*	Viperidae and Elapidae	Costa Rica, Thailand, China, Sri Lanka, India, Nigeria, Papua New Guinea	Varespladib	PLA_2_	In vitro	Varespladib effectively neutralized PLA_2_-driven coagulopathic activities and partially inhibited procoagulant effects from non-PLA_2_ toxins.	2020	[89]
54	*E. ocellatus*, *E. carinatus*, *B. asper*, *B. arietans*, *D. russelii*	Viperidae	Nigeria, India, Costa Rica, Nigeria, Sri Lanka	Nafamostat, varespladib, marimastat, dimercaprol, DMPS, batimastat	SVMP, PLA_2_, SVSP	In vitro, In vivo	Nafamostat and dimercaprol alone showed limited efficacy against whole snake venom, but in combination with varespladib and marimastat achieved broad and effective venom neutralization.	2020	[24]
55	*B. asper*, *B. jararaca*, *C. rhodostoma*, *D. acutus*	Viperidae	Costa Rica, Brazil, Thailand, China	Varespladib, dimercaprol, marimastat	PLA_2_, SVMP, SVSP CTLs	In vitro	Varespladib effectively inhibited anticoagulant effects induced by PLA_2_s. Marimastat and dimercaprol targeted several procoagulant SVMPs, but showed limited effect on anticoagulant toxins. Combinations of small molecules are likely required to achieve targeted venom neutralization.	2020	[22]
56	*C. atrox*	Viperidae	Sigma Aldrich, Dorset, UK	Batimastat, marimastat	SVMP	In vitro	Both batimastat and marimastat effectively neutralized the metalloprotease activity of purified toxin and whole venom.	2020	[32]
57	*B. atropos*, *B. caudalis*, *B. cornuta*, *B. xeropaga*	Viperidae	Africa	Varespladib	PLA_2_	In vitro	Varespladib effectively neutralized the PLA_2_-driven anticoagulant effects of prothrombinase in various *Bitis* species.	2020	[90]
58	*L. annulata*	Viperidae	Magdalena Valley, Colombia	Varespladib, EDTA, AEBSF (pefabloc)	PLA_2,_ SVMP, SVSP	In vitro, In vivo	The use of metalloproteinase inhibitors like EDTA and varespladib effectively neutralized the venom’s proteolytic and hemorrhagic effects.	2020	[91]
59	*M. laticollaris*, *M. fulvius*, *M. ibiboboca*, *M. obscurus*, *M. tener*	Elapidae	Venom samples from Australia, Mexico, Brazil, USA	Varespladib	PLA_2_	In vitro, In vivo	Varespladib completely neutralized the anticoagulant activities of all *Micrurus* venoms.	2020	[92]
60	*D. russelii*	Viperidae	Chennai, Tamilnadu, India	Varespladib	PLA_2_	In vitro, In vivo	Administering varespladib effectively restored clot formation by inhibiting PLA_2_ activity.	2020	[93]
61	*V. berus nikolskii*	Viperidae	Kharkiv region of Ukraine	Varespladib	PLA_2_	In vivo	Varespladib exhibited significant protection against envenomation, achieving 80% survival in treated mice.	2020	[94]
62	*B. jararaca*	Viperidae	Butantan Institute in São Paulo, Brazil	EDTA, pefabloc	SVMP, SVSP	In vitro, In vivo	EDTA significantly inhibited venom-induced proHGFA activation, whereas pefabloc showed no effect, identifying SVMPs as major mediators of HGF/c-Met pathway activation and elevated plasma HGF levels.	2019	[95]
63	*B. arietans*	Viperidae	South Africa	EDTA, PMSF	SVSP, SVMP	In vitro	EDTA was found to have no effect on Kn-Ba activity, whereas PMSF effectively blocked its fibrinogenolytic activity.	2018	[96]
64	*D. acutus*	Viperidae	Huangshan, China	Varespladib	PLA_2_	In vivo	Varespladib is an effective antidote, which significantly reduced local and systemic damage by inhibiting PLA_2_ activity, preventing muscle atrophy and fibrosis.	2018	[97]
65	*M. fulvius*	Elapidae	North America	LY315920 (varespladib) and LY333013 (orally bioavailable prodrug)	PLA_2_	In vivo	Varespladib and methyl-varespladib are highly effective in preventing lethality and can reverse severe neurotoxicity, coagulopathy, myotoxicity, and intravascular hemolysis even after envenoming was established.	2018	[98]
66	*A. lubricus*, *A. scuttatus*, *B. fasciatus*, *D. polylepis*, *E. boulengeri*, *E. sundevallii longicauda*, *E. s. sundevallii*, *H. haemachatus*, *N. annulata*, *N. annulifera*, *N. atra*, *N. haje*, *N. kaouthia*, *N. melanoleuca*, *N. mossambica*, *N. naja*, *N. nigricincta*, *N. nigricollis*, *N. nivea*, *N. pallida*, *N. phillippinensis*, *N. samarensis*, *N. siamensis*, *N. sumatrana*, *O. hannah*, *W. aegyptia*	Elapidae	Africa and Asia	Varespladib	PLA_2_	In vitro	Varespladib neutralized venom-induced coagulopathic effects, outperforming commercial antivenom.	2018	[99]
67	*O. scutellatus*	Elapidae	Papua New Guinea	Varespladib	PLA_2_	In vivo	Oral, heat-stable varespladib greatly improved survival and reversed presynaptic neurotoxicity even when antivenom failed to neutralize the effects.	2018	[100]
68	*E. ocellatus*	Viperidae	Latoxan, Cameroon and Ghana	Marimastat, batimastat	SVMP	In vitro, In vivo	Marimastat and batimastat effectively reduced hemorrhagic, coagulant, defibrinogenating, and proteolytic venom actions.	2017	[101]
69	*B. jararaca*	Viperidae	Brazil	EDTA, pefabloc	SVMP, SVSP	In vitro, In vivo	SVMPs are the essential toxins driving coagulopathy and hemorrhage; SVSPs contributed minimally. Crucially, neither Na_2_-EDTA nor AEBSF could prevent a drop in platelet count, proving that neither SVMPs nor SVSPs are responsible for venom-induced thrombocytopenia.	2014	[102]
70	*B. lateralis*	Viperidae	Instituto Clodomiro Picado, Costa Rica	Batimastat	SVMP	In vitro, In vivo	Batimastat completely reduced the BlatH1-induced inhibiton of platelet aggregation, showing that the anti-platelet effect is dependent on the metalloproteinase domain rather than the disintegrin-like domain.	2014	[103]
71	*V. ammodytes*	Viperidae	Institute of Immunology Inc., Zagreb, Croatia	Pefabloc EDTA	SVSP, SVMP	In vitro, In vivo	VaSP1 was found to be a novel, active anticoagulant serine protease that depletes fibrinogen and prothrombin. Studies using pefabloc and EDTA proved that VaSP1 is a catalytically active serine residue.	2013	[104]
72	*B. asper*	Viperidae	Instituto Clodomiro Picado, Costa Rica	Batimastat	SVMPs and PLA_2_	In vivo	Batimastat alone was found to be ineffective in treating extracellular matrix degradation, muscle injury, and protection against severe pathology.	2011	[105]
73	*B. asper*	Viperidae	Pacific region of Costa Rica	Batimastat	SVMP	In vitro and In vivo	Batimastat was not effective in treating myotoxic PLA_2_-dependent effects such as lymphatic vessel malfunction and edema.	2008	[106]
74	*B. asper*	Viperidae	Costa Rica	Batimastat	SVMP	In vivo	Batimastat completely prevented BAP1-induced acute hemorrhagic activity and subsequent dermonecrosis, demonstrating that long-term tissue damage is initiated by early SVMP-mediated proteolysis.	2008	[107]
75	*E. ocellatus*	Viperidae	Kaltungo, Nigeria	Marimastat, prinomastat, EDTA	SVMP	In vivo	Marimastat and prinomastat strongly inhibited venom metalloproteinases and reduced venom-induced hemorrhage. Broad-spectrum inhibition of local hemorrhage may require a synthetic MMPI mixture to target structurally diverse SVMPs.	2007	[38]
76	*B. asper*	Viperidae	Costa Rica	Batimastat	SVMP	In vivo, Ex vivo	Batimastat reduced protease-dependent platelet hypoaggregation but not thrombocytopenia.	2005	[108]
77	*B. asper*	Viperidae	Central America and Southern Mexico	Batimastat	SVMP	In vitro	BaP1 induced cell detachment and apoptosis through ECM degradation and anoikis. Batimastat prevented these effects.	2005	[109]
78	*B.asper*	Viperidae	Costa Rica	Batimastat	SVMP	In vitro, In vivo	Batimastat neutralized in vitro proteolytic, hemorrhagic, and coagulant activities when preincubated with venom, but failed to inhibit myotoxic activity or lethality when administered after envenoming.	2004	[110]
79	*B. jararaca*	Viperidae	South America	Batimastat	SVMP	In vitro, In vivo	Batimastat inhibited jararhagin-induced pulmonary hemorrhage, confirming that hemorrhagic effects are caused due to SVMP proteolytic activity.	2003	[111]
80	*B. asper*	Viperidae	Costa Rica	Batimastat	SVMP, PLA_2_	In vitro, In vivo	Batimastat did not reduce myotoxicity or edema, showing that endogenous MMPs play a limited role in venom-induced pathology.	2002	[112]
81	*T. mucrosquamatus*	Viperidae	Taiwan	Batimastat and synthetic hydroxamate analogs	SVMP	In vitro and In vivo	Structural analysis of batimastat revealed that it inhibits SVMPs by mimicking substrate transition states and coordinate catalytic Zn^2+^ ions. The S’1 pocket of the enzyme was found to be a key determinant of inhibitor potency and selectivity.	2002	[113]
82	*B. asper*	Viperidae	Costa Rica	Batimastat	SVMP	In vivo	Batimastat effectively inhibited proteolytic, hemorrhagic, and dermonecrotic effects, and partially reduced edema-forming activity. Early local injections are critical to prevent permanent tissue damage.	2000	[34]
83	*B. asper*	Viperidae	Costa Rica	Batimastat	SVMP	In vitro and In vivo	Batimastat effectively inhibited BaP1-mediated hemorrhage, dermonecrosis, and edema, especially when administered rapidly at the site of envenomation.	2000	[114]
84	*A. blomhoffii brevicaudus*	Viperidae	Kyungpook National University, Taegu, Korea	Pefabloc	SVSP	In vitro	Pefabloc completely inhibited the fibrinogenolytic activity of brevinase, ensuring its classification as a serine protease.	1999	[115]

**Figure 3:**
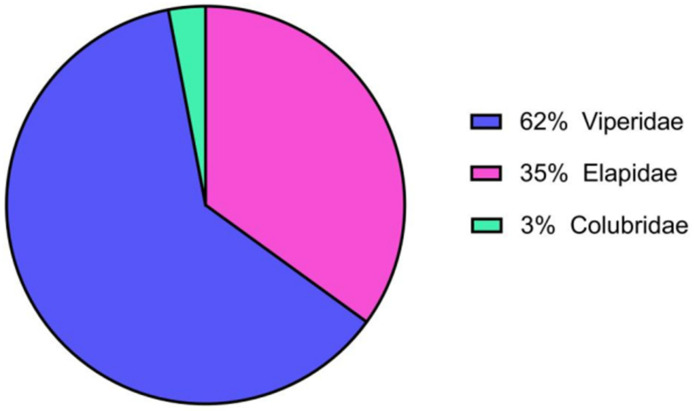
Distribution of snake families included in studies evaluating small molecule inhibitors against snake venom toxin families.

Studies investigating SMIs in snakebite research have mainly focused on venoms from the Viperidae family (62%), followed by Elapidae (35%), while only limited studies have examined Colubridae species (3%) (Figure 3). This distribution clearly shows the impact of viper envenomation, where the toxins such as PLA_2_s, SVMPs, and SVSPs contribute to severe hemorrhage, coagulopathy, local tissue destruction, and systemic toxicity. The representation of elapid venoms also reflects growing interest in compounds capable of neutralizing PLA_2_-mediated neurotoxic effects, such as the development of broad-spectrum inhibitors like varespladib. The very limited number of studies involving colubrid venoms highlights a major research gap and implies the need for more comprehensive studies to understand how widely SMI-based therapies can be applied across snakebite envenomation.

## 3. Use of Small Molecule Inhibitors as Antidotes Against Major Enzymatic Proteins in Snake Venom

Snake venom is a cocktail of bioactive proteins, peptides, organic, and inorganic constituents that are highly effective in inducing various pharmacological effects post-envenomation. Among these, several enzymatic and non-enzymatic proteins constitute the major portion of snake venom. Depending on the relative abundance, venom proteomics have shown that snake venom proteins fall under major and minor categories and PLA_2_, SVSP, SVMP, and three-finger toxins represent the major snake venom protein families [116,117]. These proteins not only disrupt hemostasis and neuromuscular function but also trigger inflammatory and immunomodulatory responses that contribute to local and systemic pathology post-envenomation [118]. In viperid venoms, SVMP, SVSP, and PLA_2_ enzymes play major roles in hemorrhage, coagulopathy, tissue injury, edema, and inflammatory damage, whereas elapid venoms are dominated by three-finger toxins and PLA_2_s, which are more closely linked to neurotoxicity and related systemic effects [117].

### 3.1. Phospholipase A_2_

PLA_2_ enzymes are among the most abundant and important toxins in snake venom, particularly because they drive inflammation through both catalytic and non-catalytic mechanisms [119]. They hydrolyze glycerophospholipids at the sn-2 position of the glycerol backbone, resulting in the release of phospholipids and free fatty acids. Snake venom PLA_2_ (PLA_2_s) exhibits considerable sequence similarity, sharing nearly 44–99% amino acid identity, which contributes to their highly conserved three-dimensional structure. Based on factors such as molecular size, localization, biological function, substrate specificity, and calcium dependency, PLA_2_ enzymes are categorized into six major families. PLA_2_s belong primarily to the secretory PLA_2_ (PLA_2_) family, particularly groups IA, IIA, and IIB. Group IA PLA_2_s are most common in elapid snake venoms, while group IB PLA_2_s are less common. In viper venoms, group IIA PLA_2_s are the main type, and group IIB PLA_2_s are found only rarely [120]. PLA_2_s stimulate inflammatory signaling through NF-κB activation, cytokine release including TNF-α, IL-1β, and IL-6, leukocyte recruitment, mast cell degranulation, and macrophage activation, thereby amplifying both local and systemic pathology after envenomation [118]. Because these toxins contribute not only to local inflammation and necrosis but also to anticoagulant, neurotoxic, and other systemic effects, PLA_2_s have become major therapeutic targets in snakebite research, with inhibitors such as varespladib showing promise as adjunct antidotes [121].

The studies summarized in Table 1 demonstrate the broad-spectrum efficacy of varespladib in inhibiting venom PLA_2_ activity and neutralizing PLA_2_-mediated toxic effects across diverse snake species. Varespladib was first reported as a snake venom inhibitor in a 2016 study by Lewin et al., who showed potent inhibition of PLA_2_ activity across 28 medically important snake venoms. A further initial in vivo protection was demonstrated against lethal envenoming by *Micrurus fulvius* and *Vipera berus* [26]. Varespladib is effective in neutralizing PLA_2_-driven toxic effects and delaying lethality in preclinical models. In addition to that, it has also been shown to restore or reduce toxic manifestations, which could result in significantly better recovery rates and reduced long-term complications. In several cases, the efficacy of varespladib exceeds that of the available antivenom, specifically in inhibiting the PLA_2_-mediated toxic effects of envenomation. In African spitting cobra venoms, SAIMR polyvalent antivenom showed little or no neutralization of the venom-induced anticoagulant activity, whereas varespladib effectively inhibited the PLA_2_-mediated disruption of thrombin and factor Xa function [99]. In Chinese *Daboia siamensis* venom, varespladib was more effective than antivenom in reversing established neurotoxic and myotoxic effects after venom exposure [62]. Similarly, in Central American pit vipers, PolyVal-ICP antivenom was less effective than varespladib against the venoms of *Metlapilcoatlus mexicanus* and *M. nummifer*, where varespladib provided better protection against twitch inhibition and myotoxicity. In studies involving *Daboia russelii* venom, varespladib significantly reduced venom-induced coagulopathy and restored normal clot formation by inhibiting PLA_2_-mediated fibrinogen degradation. When combined with marimastat, it further improved protection by reducing alterations in PT and aPTT and enhancing survival even when treatment was delayed [47]. The inhibitor also showed remarkable efficacy against *Deinagkistrodon acutus* venom by preventing muscle atrophy and fibrosis, thereby promoting improved functional recovery following envenomation [97]. Further studies have highlighted the potential of varespladib to neutralize PLA_2_ and PLA_2_-like toxins from medically important snakes of the *Bungarus* species, where varespladib rescued mice from fatal neurotoxicity in a post-envenomation rescue model, supporting its efficacy against presynaptic PLA_2_-mediated paralysis [73,74]. Ex vivo and in vivo studies have shown that varespladib effectively prevents or reverses venom-induced neuromuscular blockade and acute neurotoxicity, although differences in venom potency and dose requirements have been noted among species. In juvenile pig models of Australian and Papuan *Oxyuranus scutellatus* envenoming, both intravenous and oral methyl-varespladib reduced the severity of clinical weakness, reversed established neurotoxic signs, and prevented death [64]. Also, in a murine rescue model, Gutiérrez et al. showed that varespladib and methyl-varespladib retained efficacy even when administered after envenomation by several presynaptically neurotoxic venoms from *Notechis scutatus*, *Crotalus durissus terrificus*, *Bungarus multicinctus*, and *Oxyuranus scutellatus*, although the degree of protection varied by species, demonstrating their potential as effective post-envenomation therapeutic agents capable of reducing both local and systemic venom-induced toxicities [27].

Varespladib should not be described simply as a broadly effective PLA_2_ inhibitor, because its actual benefit depends on how strongly PLA_2_ toxins contribute to the pathology of a particular venom and in what structural form those toxins occur [25]. In crude venoms, PLA_2_ toxins may be present as free enzymatic toxins, as catalytically inactive PLA_2_-like homologues, or as part of multi-subunit neurotoxic complexes such as β-bungarotoxin, in which the PLA_2_ subunit is linked to a Kunitz-type partner [27]. This means that inhibition of PLA_2_ activity does not automatically guarantee full neutralization of whole-venom toxicity. Rescue studies have shown that varespladib can act against PLA_2_ toxins across different quaternary structures, including monomeric notexin, dimeric crotoxin, β-bungarotoxin, and trimeric taipoxin, but the degree of protection varies, showing that toxin complex formation, venom composition, and timing of treatment all influence outcome [27]. Structural studies further strengthen this interpretation by showing that varespladib can also bind catalytically inactive PLA_2_-like toxins, indicating that its action is not limited only to blocking enzymatic hydrolysis [122]. At the same time, these findings also define an important limitation: even when varespladib effectively inhibits a PLA_2_ toxin or PLA_2_-like component, it does not guarantee control of the overall envenoming syndrome, because other toxin families, including three-finger toxins and metalloproteinases, may continue to drive pathology [25]. A systematic comparison of the literature also shows that not all study designs are equally informative, since preincubation assays may overestimate therapeutic potential, whereas rescue models are much closer to real snakebite treatment [123]. The same caution applies to outcome measures, because benefit against neurotoxicity, coagulopathy, myotoxicity, or local tissue damage does not necessarily predict equal benefit against all other toxic effects [4]. Evidence from combination studies further suggests that varespladib alone may be insufficient in venoms with complex toxin mixtures, and that pairing it with metalloproteinase inhibitors can provide broader protection than monotherapy [47]. Oral methyl-varespladib has therefore been proposed as a possible prehospital or pre-referral adjunct because of its oral bioavailability and early toxin-directed mechanism, but current evidence does not support it as a standalone field treatment or a replacement for antivenom [25,28]. This is reflected in the BRAVO Phase II trial, where oral varespladib was safe but did not meet the primary endpoint in the overall study population, although early treatment showed a possible signal of benefit [61]. Overall, the key unresolved question is no longer whether varespladib can inhibit venom PLA_2_s, but under which venom profiles, toxin assemblies, treatment delays, and clinical settings it can provide meaningful benefit as an adjunct to antivenom therapy.

### 3.2. Snake Venom Metalloproteases

SVMPs are zinc-dependent endopeptidases predominantly found in viper venoms and play a central role in the pathophysiology of snakebite envenomation [124]. These toxins belong to the M12 family of metalloproteases, which also includes the ADAM (a disintegrin and metalloproteinase) proteins [125]. Based on their domain organization, SVMPs are structurally classified into four major groups: (i) P-I, containing only a metalloproteinase domain, (ii) P-II, containing a metalloproteinase domain and a disintegrin domain, (iii) P-III, composed of metalloproteinase, a disintegrin-like, and a cysteine-rich domain, and (iv) P-IV, featuring the P-III domain structure along with lectin-like domains [126]. Among these, P-III SVMPs exhibit significantly higher hemorrhagic activity than other subgroups in humans [127]. SVMPs contribute extensively to both local and systemic toxic effects following a snakebite. Their primary mechanism involves proteolytic degradation of fibrin and fibrinogen, leading to defibrinogenation of blood, lysis of fibrin clots, and a decrease in the blood viscosity [128]. SVMPs also play a major role in triggering inflammatory responses. They induce edema formation, recruitment of leukocytes into the tissues, neutrophil activation and migration, cytokine release, coagulation activation, and formation of neutrophil extracellular traps [119,129]. SVMPs are major mediators of venom-induced hemorrhage due to their ability to degrade basement membrane and extracellular matrix proteins, resulting in disruption of microvessel integrity, capillary damage, and blood extravasation [128,130,131,132].

Conventional antivenom alone was found to be ineffective against SVMP-induced hemorrhage and tissue damage [133,134,135]. As a result, a growing number of studies have sought to identify more effective inhibitors or modulators of these SVMP-mediated activities. Experimental studies demonstrate that inhibition of SVMP activity markedly reduces venom toxicity, confirming SVMPs as critical therapeutic targets in viper envenomation [132,136]. Among the investigated inhibitors, marimastat and batimastat showed potent and broad-spectrum SVMP neutralization across several medically important snake species. These inhibitors effectively blocked hemorrhagic, fibrinogenolytic, proteolytic, procoagulant, and dermonecrotic activities both in vitro and in vivo [32,34,101,108,114]. Batimastat was particularly effective in preventing local tissue damage when administered rapidly at the bite site, although its efficacy decreased with delayed or systemic administration [101,114]. Marimastat emerged as one of the most promising SVMP inhibitors due to its high potency, broad-spectrum activity, and effectiveness against venom-induced coagulopathy [44,50,53]. Several studies demonstrated that marimastat outperformed metal chelators such as DMPS and dimercaprol in neutralizing SVMP-mediated toxicities [22,24,44]. Prinomastat also exhibited strong inhibitory activity, particularly against venom-induced factor X activation in procoagulant venoms such as *Daboia* and *Macrovipera* [87,88]. The available evidence indicates that the efficacy of SMIs depends on venom composition, and that no single inhibitor is likely to provide universal protection. In *Bitis* venoms, varespladib most effectively neutralized PLA_2_-driven anticoagulant activity, whereas marimastat and prinomastat were less effective, and none of the inhibitors blocked the pseudo-procoagulant activity of *B. caudalis*. Likewise, in *Echis ocellatus*, metalloprotease inhibitors and metal chelators differed in their ability to inhibit hemorrhagic activity, highlighting the need for toxin-targeted therapeutic strategies [38,78]. Cross-neutralizing effects among inhibitors have also been reported, with varespladib and prinomastat showing overlapping inhibitory activity against certain venom toxins. Chowdhary et al. demonstrated that the metalloprotease inhibitor prinomastat inhibited FXa-blocking PLA_2_ toxins of all African spitting cobras (*Naja* species) (Table 1) at the same concentration (2 mM) used for inhibiting metalloproteases, making it comparably effective in cross-reactivity against both toxin families. The study also showed that varespladib and the metalloprotease inhibitors marimastat and prinomastat exhibited cross-reactivity, with prinomastat being significantly more effective than marimastat in reducing PLA_2_-driven anticoagulant toxicity [86]. Despite this broader activity, prinomastat displayed variable efficacy across venom-induced pathologies, effectively neutralizing tissue damage caused by *Daboia russelii* venom but showing limited protection against cobra venom-induced hemorrhage [60]. Another study [79] demonstrated that both marimastat and prinomastat inhibited the procoagulant activity of *Atheris*, *Cerastes*, and *Proatheris*, with marimastat consistently showing greater potency than prinomastat across all tested species [79].

EDTA has been widely used as a mechanistic probe for SVMP-dependent toxicity because it chelates the catalytic Zn^2+^ required for metalloprotease activity, and can also disrupt structural divalent metal support. EDTA appears to have been adopted in snake venom research by at least 1960 in work on Russells viper venom, with a further study in 1961 on Habu venom, indicating that metal chelation was recognized early as a means of attenuating venom toxicity [137,138]. In crude venoms such as *Bothrops jararaca* and *Leptodeira annulata*, as well as in purified toxins such as Pictolysin-III from *Bothrops pictus*, EDTA suppresses proteolysis, fibrinolysis, hemorrhage, and related downstream pathologies, thereby confirming the central contribution of SVMPs to these effects [65,91,102]. Additionally, unithiol, a metal-chelating inhibitor targeting snake venom metalloproteases, has entered Phase I clinical testing and was shown to be well-tolerated in healthy Kenyan adults, with no serious adverse reactions or dose-limiting toxicities reported [40].

Current studies on SVMP inhibitors show that, although these compounds are promising, each still has important limitations that prevent it from being viewed as a universal solution for viper envenoming [24]. Batimastat showed strong inhibition of hemorrhage and local tissue damage in experimental models, but its benefit was greatest when given very early and locally, and it appears much less practical for delayed or systemic treatment, which limits its real clinical use. Marimastat is currently the most attractive SVMP inhibitor because it is potent, broadly active, and more drug-like than batimastat, but even marimastat should not be presented as sufficient on its own, since it does not neutralize other major toxin families and often performs best when combined with a PLA_2_ inhibitor such as varespladib [24]. This is especially clear in venoms containing multiple medically important toxin families, where blocking SVMPs may reduce one major pathology but still leave coagulopathy, cytotoxicity, or lethality driven by other toxins unresolved [24]. Prinomastat also appears useful in some models, including inhibition of venom procoagulant activity [87], but the evidence for it is still less extensive and its efficacy seems to vary depending on the venom and the pathological endpoint being measured, suggesting that its apparent cross-reactivity does not necessarily translate into equally broad therapeutic protection. Metal chelators such as EDTA have been very useful for showing that SVMPs are major drivers of hemorrhage and proteolysis [102], but EDTA is better viewed as a mechanistic laboratory tool than a realistic therapeutic candidate because its chelating action is non-selective and not tailored for routine snakebite treatment. Unithiol/DMPS is more clinically interesting because it is already an approved chelator and has shown early intervention benefit in preclinical studies, yet it also has clear limits [67,79]. It is mainly effective against metal-dependent toxins, its efficacy varies across species, protection may fall with time because repeat dosing may be needed, and it still cannot replace antivenom in venom where non-SVMP toxins contribute strongly to overall toxicity. It should be noted that SVMP inhibitors have been studied mainly against viperid venoms rather than elapid venoms, which is biologically expected because SVMPs are major toxin components in many viperid venoms, whereas elapid venoms are more often dominated by other toxin families such as three-finger toxins and PLA_2_s [24,116]. Accordingly, most therapeutic interest in SVMP inhibition has focused on hemotoxic and tissue-damaging viper envenoming, where these toxins are more directly linked to pathology. Overall, these studies suggest that the main challenge for current SVMP inhibitors is not lack of inhibitory potency, but their ability to provide consistent therapeutic protection across diverse venoms, particularly when treatment is delayed and multiple toxin families contribute to venom pathology. These findings further highlight the potential need for combination therapies rather than SVMP-targeted monotherapy [47].

### 3.3. Snake Venom Serine Proteases

SVSPs represent one of the major enzymatic toxin families predominantly found in viperid snakes, including members of the subfamilies Viperinae and Crotalinae [96], where they contribute primarily to venom-induced consumption coagulopathy, defibrinogenation, hemorrhage, edema, and hypotensive shock [91,139,140]. These enzymes exhibit high substrate specificity and mimic human physiological processes of the coagulation cascade and kallikrein–kinin system. Structurally, SVSPs are single-chain glycoproteins with a relative molecular mass ranging from 26 to 33 kDa, though high degrees of glycosylation can result in stable isoforms reaching up to 67kDa (e.g., Bothrops protease A). They belong to the S1 family of chymotrypsin, featuring a highly conserved catalytic triad composed of His43, Asp88, and Ser184. Despite sharing high sequence similarity among themselves, SVSPs exhibit significantly lower identity with human thrombin (26–33%) or plasma kallikrein (34–40%), which contributes to their functional divergence and resistance to human serpins (serine protease inhibitors) [96,139]. Rare heterodimeric forms, such as brevinase, also exhibit high thermal and pH stability [115].

SVSPs exert toxicity through multiple mechanisms including fibrinogen degradation, thrombin-like activity, activation of coagulation factors, and kinin release [141]. Some SVSPs act directly upon fibrinogen, typically cleaving the α and β chains, leading to defibrinogenation and impaired clot formation. While some thrombin-like enzymes produce weak, friable fibrin clots (pseudo-procoagulant activity), others, like *Bothrops jararaca* Serine Protease (BjSP) degrade fibrinogen without forming any clot at all, leading to anticoagulation through fibrinogen depletion [139]. SVSPs promote the release of bradykinin, which induces vasodilation and increases vascular permeability, potentially leading to severe hypotension and fatal shock [96].

Traditional antivenoms often fail to fully neutralize SVSP-mediated proteolytic activity in in vitro conditions [133,139,142], highlighting an important therapeutic gap. Several SMIs targeting SVSPs have been investigated and summarized in Table 1. Classical serine protease inhibitors such as pefabloc and nafamostat have been widely employed not only to inhibit SVSP activity, but also to characterize and confirm the identity of venom enzymes [96]. Nafamostat has shown potent in vitro inhibition of SVSP activity in a dose-dependent manner against venoms from *E.carinatus*, *Bothrops asper*, and *Bitis arietans*. Despite potent in vitro activity, nafamostat is unsuitable for snakebite treatment due to its short half-life, requirement for intravenous administration, off-target anticoagulant interactions, and inability to protect against venom-induced lethality in murine models [24]. Likewise, inhibition of SVSPs with pefabloc failed to significantly improve defibrinogenation or thrombocytopenia in certain envenomation models, indicating that SVSPs may only have a limited contribution to these systemic disturbances compared with other toxin families [102]. AEBSF/Pefabloc, an irreversible inhibitor of serine proteases [115], effectively inhibited fibrinogenolytic activities in several viper venoms. They are not the primary drivers of coagulopathy in venoms like *B. jararaca*, where the coagulant activity was unaffected by AEBSF but inhibited by SVMP inhibitors [82,102]. SVSPs affect inflammation in several ways. For instance, some SVSPs including Cdtsp2 from *Crotalus durissus terrificus* promote edema formation through activation of protease-activated receptors (PAR1 and PAR2), and Kn-Ba from *Bitis arietans* induces inflammatory responses by releasing cytokines [143,144], while others, such as FII and asperase, were found to possess immunosuppressive properties [145,146]. Additionally, certain SVSPs can exhibit biological activities without altering the inflammatory pathways [147]. Nevertheless, studies indicate that SVSPs are often less critical to immediate lethality than SVMPs and PLA_2_s in many viperid venoms [24].

Although SVSP inhibitors have demonstrated effective inhibition of their target enzymes in biochemical assays, their ability to provide protection against the complex pathological effects of whole venoms remains limited. Nafamostat is a clear example of this limitation. Although it effectively inhibits SVSP activity in vitro [44], this has not translated into significant protection against venom-induced lethality. Furthermore, its inclusion in inhibitor combinations containing SVMP and PLA_2_ inhibitors has not always resulted in improved therapeutic efficacy against certain viper venoms [20]. Its short half-life, need for intravenous administration, and potential to interfere with normal coagulation also make it a difficult candidate for practical snakebite treatment [4]. Similar limitations have been reported for pefabloc. Although it is useful for confirming or characterizing serine protease activity, inhibition of SVSP-mediated fibrinogenolysis alone does not necessarily improve the overall manifestations of envenomation [102]. In many venoms, coagulopathy, hemorrhage, and lethality are driven predominantly by SVMPs, PLA_2_s, or the combined action of multiple toxin families [148,149,150]. This may explain why several SVSP inhibitors show promising activity in enzymatic assays but demonstrate limited efficacy in whole-venom models. The contribution of SVSPs to venom pathology varies considerably among species, and even in venoms with substantial SVSP content, clinical effects often arise from the synergistic action of multiple toxins rather than SVSPs alone. Another important limitation is that many studies continue to rely on in vitro assays or venom-inhibitor preincubation experiments, which may overestimate therapeutic efficacy compared with post-envenomation rescue models that more closely mimic real-world snakebite scenarios [44,104,115]. Collectively, the available evidence suggests that while SVSPs remain attractive therapeutic targets, the development of clinically useful SVSP inhibitors will require compounds with improved selectivity, a longer duration of action, and validation in clinically relevant rescue models. Future studies should also determine whether targeting SVSPs provides additional therapeutic benefit when combined with established strategies aimed at SVMPs and PLA_2_s.

## 4. Discussion

Although antivenoms are still the main treatment for snakebite envenoming, they have several important limitations [4,151]. In many rural areas, patients do not receive antivenom quickly enough, and treatment usually requires intravenous administration in a hospital or well-equipped healthcare building. Antivenoms also do not always work equally against venoms from different snake species or from different geographical regions, and they are often less effective against rapidly developing local tissue damage and some early toxin-induced effects [133]. In addition, their wide use is limited in many high-burden regions by problems related to cost and availability. Because of these limitations, several adjunct strategies are now being explored to improve snakebite treatment. Among these, small molecule inhibitors are especially attractive because they can target conserved toxin families that are directly responsible for early morbidity and mortality. In the toxin families reviewed here, PLA_2_s and SVMPs are currently the most promising targets, whereas SVSPs appear to be less suitable as primary therapeutic targets in many venoms. Varespladib has shown broad activity against PLA_2_-mediated toxic effects, including anticoagulant and neurotoxic actions, while SVMP inhibitors marimastat, batimastat, prinomastat, and certain metal chelators have shown useful effects against hemorrhage, coagulopathy, and tissue damage. In some cases, combining inhibitors, such as varespladib with marimastat, has produced broader protection than monotherapy, suggesting that multi-target approaches may be more realistic than relying on a single inhibitor. At the same time, the literature shows that these inhibitors are not yet a complete solution. A major limitation is that most of them are highly toxin-specific, which means that they may work well against one toxin family but leave other clinically important toxins unaffected. Their effects may therefore be incomplete in venoms that contain complex mixtures of toxins, and the same inhibitor may not work equally well across different snake species, populations, or regions. Another important limitation is the chosen methodology, meaning that many studies still rely on in vitro and preincubation models where venom and inhibitor are mixed before administration, even though these conditions do not fully reflect human envenoming where venom has already entered tissues and started causing damage before treatment begins. This means that some published results may overestimate the real therapeutic value of certain inhibitors, and more rescue-based studies are needed [133]. Beyond small molecule inhibitors, new biologic adjuncts are also being developed, including recombinant monoclonal antibodies, oligoclonal antibody cocktails, and nanobody-based therapies, which may provide more defined toxin targeting, better batch consistency, and potentially fewer adverse reactions than conventional animal-derived antivenoms [24]. These approaches are particularly attractive for toxins that are poorly neutralized by current antivenoms, such as some three-finger toxins and cytotoxins. Recent work on nanobody-based recombinant antivenoms has shown broad protection against African elapid venoms and better reduction in local tissue damage in some rescue models, suggesting that such biologics may eventually complement or improve conventional antivenom therapy [152]. Other early-stage approaches include aptamer-based inhibitors, which are being explored mainly against cobra cytotoxins, especially for local tissue injury that antivenoms often fail to control well [153]. In addition to direct toxin binders, there are also symptomatic or mechanism-based adjuncts such as neostigmine, which can improve neuromuscular transmission in some neurotoxic bites, and other repurposed agents that may support recovery after neurotoxic injury [4]. However, most of these alternatives are still at an early stage, and major problems remain, including limited venom coverage, need for toxin-specific design, cost of development, and lack of clinically relevant rescue studies. Better pharmacokinetic and pharmacodynamic studies are also required to determine effective doses, treatment windows, and whether repeat dosing is necessary, and the safety of combining multiple agents still needs careful evaluation. Overall, both small molecule inhibitors and newer biologic or supportive adjuncts represent important and promising directions in snakebite therapeutics, but at present they should be viewed as complements to antivenom rather than replacements. More clinically relevant translational studies are still needed before they can be used reliably across diverse snakebites.

## 5. Conclusions and Future Perspectives

Despite the availability of conventional antivenoms, snakebite envenoming remains a major neglected tropical disease associated with significant mortality, morbidity, and long-term disability. Increasing evidence shows that major snake venom enzymatic proteins, such as snake venom metalloproteinases, phospholipase A_2_s, and serine proteases, play important roles in causing both local and systemic pathology. Repurposed small molecule inhibitors have therefore emerged as promising adjuncts to antivenom therapy by directly neutralizing the catalytic activities of these toxins and may help reduce tissue damage, coagulopathy, hemorrhage, and other venom-induced complications. Amongst the most promising candidates are inhibitors of PLA_2_s and metalloproteinases that have shown preclinical potential and are moving towards clinical evaluation. However, the diverse nature of snake venom suggests that no single inhibitor is likely to provide universal protection, and it is more likely that broad-spectrum or multi-target inhibitors will need to be used in combination with conventional antivenoms to achieve complete neutralization. Advances in venomics, antivenomics, structural biology, and artificial intelligence are expected to accelerate the identification of clinically relevant toxin targets and design inhibitors in an efficient manner. Specifically, the development of orally bioavailable, heat-stable inhibitors for early administration in resource-limited settings where delays in access to antivenom is a significant problem that needs to be addressed. In future, clinical studies are needed to determine the safety, efficacy, and optimal use of repurposed inhibitors as adjunctive treatments in snakebite cases. In addition, new trends like nanomedicine, rapid venom diagnostics, and precision medicine could help in increasing the efficiency of treatment. These advancements collectively have the potential to revolutionize the treatment of snakebites from antivenom to integrated therapies that might reduce mortality and morbidity in snakebite cases.

## Data Availability

Not applicable.
